# Supplementary material for: Neural resources shift under Methylphenidate: A computational approach to examine anxiety-cognition interplay
Source: Neuroimage. Author manuscript; Available in PMC 2022 Dec 22. (PMC9772074; doi:10.1016/j.neuroimage.2022.119686)
Supplement: MMC1 [file NIHMS1848690-supplement-MMC1.docx]

Neural resources shift under Methylphenidate: a computational approach to examine anxiety-cognition interplay

Manish Saggar, Ph.D.^1^*^¶^, Jennifer Bruno, Ph.D.^1¶^, Claudie Gaillard, Ph.D.^2^, Leonardo Claudino, Ph.D.^2^, Monique Ernst, M.D., Ph.D.^2^*

^1^Department of Psychiatry and Behavioral Sciences, Stanford University, Stanford, CA, USA

^2^Section on Neurobiology of Fear and Anxiety, National Institute of Mental Health, Bethesda, MD, USA

**Running title:** Neural resources shift under Methylphenidate.

*Corresponding authors:

Manish Saggar, Ph.D.,

401 Quarry Rd, St 1356,

Stanford, CA USA 94305

Ph: 650-723-3656

Email: [saggar@stanford.edu](mailto:saggar@stanford.edu)

Monique Ernst, M.D., Ph.D.,

15K North Drive

Bethesda MD, 20892

Off: 301-402-9355

Email: ernstm@mail.nih.gov

^¶^ Equal contribution

**Supplementary Information**

**Supplementary Information**


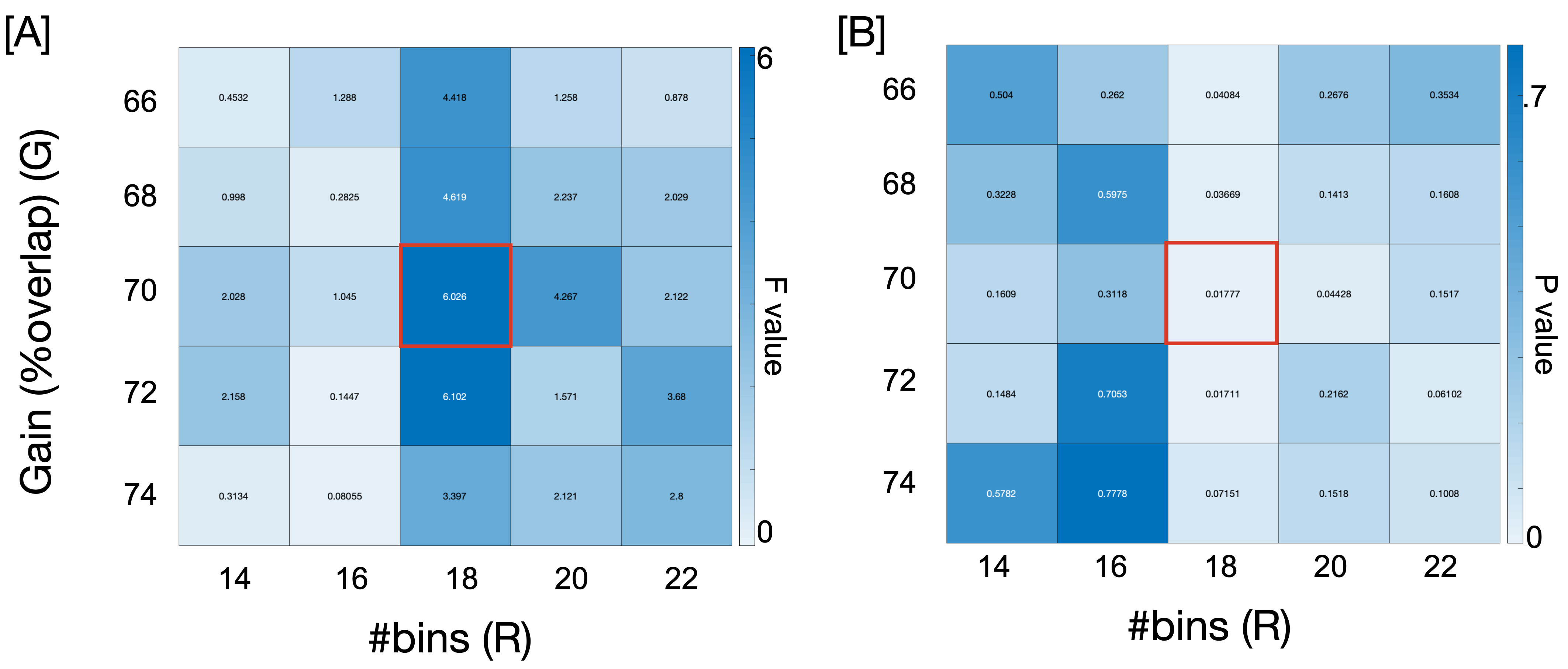


**Fig. S1:** Parameter perturbation for Mapper analysis. We varied two main Mapper parameters Gain (or %overlap between bins) and Resolution (or number of bins) to make sure results were stable across wide parameter choices. The initial values (G=70%, R=18) were chosen based on previous work. As expected, varying both Mapper parameters provide relatively similar results. [A-B] F- and p-values for estimating group differences (from one-way ANOVA) using load-based annotation on the Mapper-generated graphs.

**Fig. S2:** Mapper-generated graphs colored (annotated) by WM load for all participants in the MPH group

**Fig. S3:** Mapper-generated graphs colored (annotated) by anxiety for all participants in the MPH group

**Fig. S4:** Mapper-generated graphs colored (annotated) by WM load for all participants in the PLA group

**Fig. S5:** Mapper-generated graphs colored (annotated) by anxiety for all participants in the PLA group


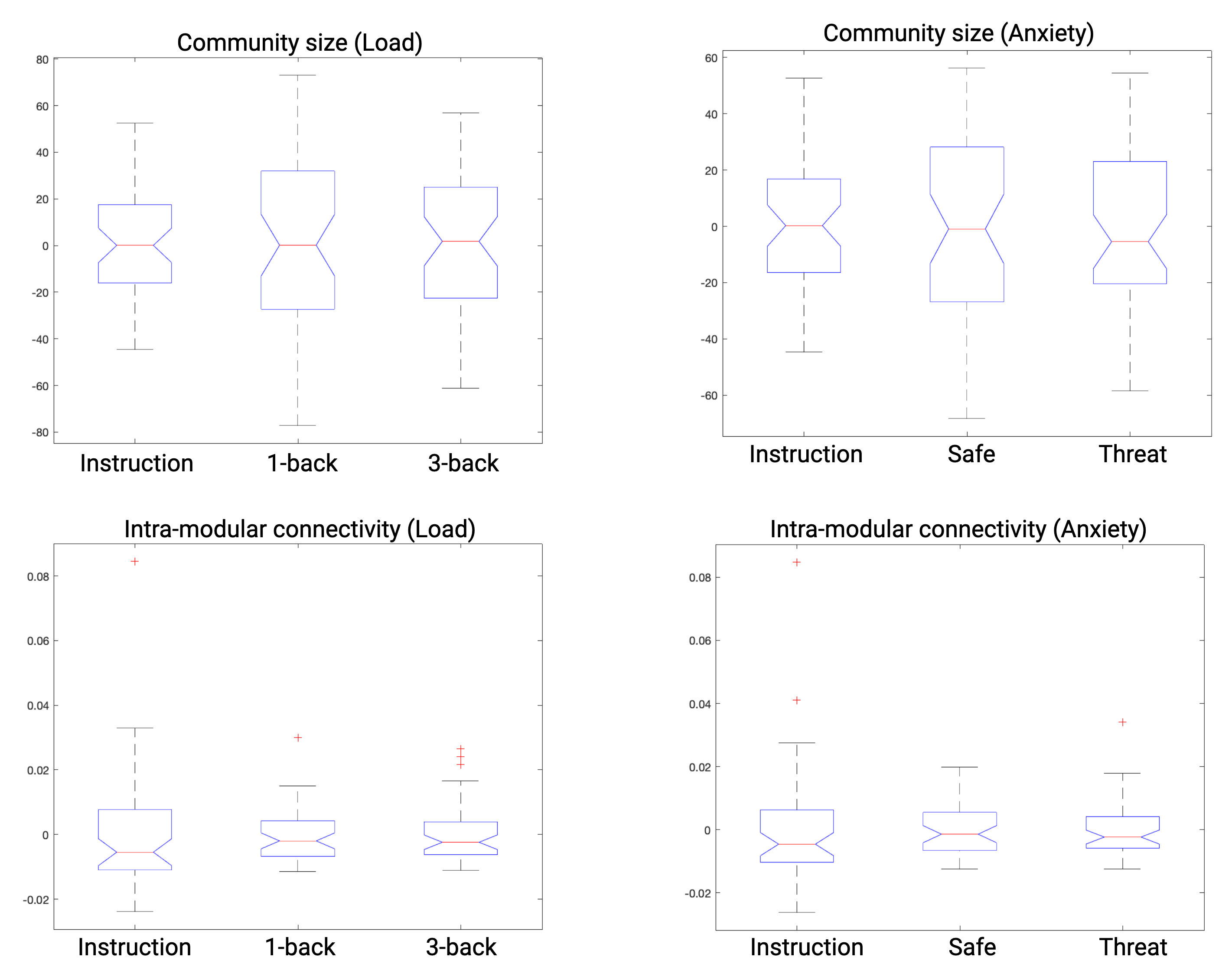


**Fig S6:** Comparing community size and intramodular connectivity across the three sub-conditions for load (i.e., instructions, 1-back and 3-back trials) and anxiety (i.e., instructions, safe and threat trials), while controlling for age and sex. No significant differences were found in community size or intramodular connectivity across sub-conditions (ps > 0.05).
